# Navigating through the Controversies and Emerging Paradigms in Early Detection of Prostate Cancer: Bridging the Gap from Classic RCTs to Modern Population-Based Pilot Programs

**DOI:** 10.3390/jpm13121677

**Published:** 2023-12-01

**Authors:** Juan Gómez Rivas, Renée C. A. Leenen, Lionne D. F. Venderbos, Jozien Helleman, Irene de la Parra, Vera Vasilyeva, Jesús Moreno-Sierra, Partha Basu, Arunah Chandran, Roderick C. N. van den Bergh, Sarah Collen, Hein Van Poppel, Monique J. Roobol, Katharina Beyer, on behalf of the PRAISE-U Consortium

**Affiliations:** 1 Department of Urology, Health Research Institute, Hospital Clinico San Carlos, 28040 Madrid, Spain; irene.delaparra@salud.madrid.org (I.d.l.P.); dr_jmoreno@hotmail.com (J.M.-S.); 2 Department of Urology, Erasmus MC Cancer Institute, University Medical Center, 3015 GD Rotterdam, The Netherlands; r.leenen@erasmusmc.nl (R.C.A.L.); l.venderbos@erasmusmc.nl (L.D.F.V.); j.helleman@erasmusmc.nl (J.H.); roodvdb@hotmail.com (R.C.N.v.d.B.); m.roobol@erasmusmc.nl (M.J.R.); k.beyer@erasmusmc.nl (K.B.); 3 European Association of Urology, Guidelines Office, PO Box 30016 6803 AA Arnhem, The Netherlands; v.vasilyeva@uroweb.org (V.V.); s.collen@uroweb.org (S.C.); 4 European Association of Urology, EAU Policy Office, PO Box 30016 6803 AA Arnhem, The Netherlands; hendrik.vanpoppel@kuleuven.be; 5 International Agency for Research on Cancer, World Health Organization, 69366 Lyon, France; basup@iarc.who.int (P.B.); chandrana@iarc.who.int (A.C.); 6 Department of Urology, Sint Antonius Hospital, 3543 AZ Utrecht, The Netherlands; 7 Department of Urology, KU Leuven, 3000 Leuven, Belgium

**Keywords:** early detection, PRAISE-U, prostate cancer, screening, overdiagnosis, overtreatment

## Abstract

Over the last three decades, the European Randomized Study of Screening for Prostate Cancer (ERSPC) and the US-based Prostate, Lung, Colorectal and Ovarian (PLCO) Cancer Screening have steered the conversation around the early detection of prostate cancer. These two randomized trials assessed the effect of screening on prostate cancer disease-specific mortality. Elevated PSA levels were followed by a systematic sextant prostate biopsy. Standard repeat testing intervals were applied. After controversies from 2009 to 2016 due to contradicting results of the two trials, the results aligned in 2016 and showed that early PSA detection reduces prostate cancer-specific mortality. However, overdiagnosis rates of up to 50% were reported, and this sparked an intense debate on harms and benefits for almost 20 years. The balance between harms and benefits is highly debated and has initiated further research to investigate new ways of early detection. In the meantime, the knowledge and tools for the diagnostic algorithm improved. This is a continuously ongoing effort which focuses on individual risk-based screening algorithms that preserve the benefits of the purely PSA-based screening algorithms, while reducing the side effects. An important push towards investigating new techniques for early detection came from the European Commission on the 20th of September 2022. The European Commission published its updated recommendation to investigate prostate, lung, and gastric cancer early detection programs. This opened a new window of opportunity to move away from the trial setting to population-based early detection settings. With this review, we aim to review 30 years of historical evidence of prostate cancer screening, which led to the initiation of the ‘The Prostate Cancer Awareness and Initiative for Screening in the European Union’ (PRAISE-U) project, which aims to encourage the early detection and diagnosis of PCa through customized and risk-based screening programs.

## 1. Introduction

Prostate cancer (PCa), a disease often characterized by its indolent nature and often also its potential lethality, continues to pose significant challenges to the medical community. The advent of prostate-specific antigen (PSA) screening ushered in an era of early detection, promising better prognoses and enhanced survival rates. However, this progress was not without its controversies. While promoting invasive tests to diagnose PCa in its early stages, these screening tests also introduced a complex ethical dilemma: the risk of overdiagnosis and overtreatment [1,2].

The controversy surrounding PCa screening has deepened over the years, fuelled by a growing body of evidence that questions the overall effectiveness of widespread screening programs. Elevated PSA levels do not always equate to aggressive, life-threatening cancer, leading to many false positives, one of the main causes of which is benign prostatic hyperplasia. These false alarms often trigger invasive procedures, such as biopsies, surgery and radiation to the prostate, which, in retrospect, might have been unnecessary. The potential physical and psychological repercussions of these interventions have given rise to a critical revaluation of screening guidelines. Moreover, societal perspectives on health, patient autonomy, and informed decision making have further complicated the issue. Men are now active participants in their healthcare journeys, asking for medical explanations and a voice in decision making. Balancing the need for early cancer detection with the preservation of person-centered care and the prevention of unnecessary medical interventions is a challenging task [3,4].

In this context, healthcare professionals and policymakers grapple with crucial questions: what screening strategies offer the most accurate results? How can we distinguish between indolent and aggressive forms of PCa effectively? How can we reduce opportunistic testing? How can we balance resources without overburdening the healthcare systems? These inquiries are dominating the current discussions while aiming to strike the delicate balance between timely diagnosis and judicious management [5].

This narrative review aims to discuss the PCa screening controversy, while presenting new solutions. We delve into the controversies of the last three decades of PCa screening by exploring historical perspectives, current research findings, ethical considerations, and healthcare policies. By examining the multidimensional aspects of this issue, we seek not only to understand the debates but also to illuminate potential paths forward. We hope to raise awareness by contributing valuable insights to the ongoing discourse, guiding future practices, policies, and ethical frameworks in early PCa detection.

## 2. Evidence Acquisition

Our narrative review aimed to comprehensively explore the current state of knowledge surrounding the early detection of PCa. The objective was to synthesize the existing literature, identify key themes, and discuss the implications for bridging the gap from classic randomized control trials to modern population-based pilot programs.

A literature search was conducted across major databases, including PubMed, Google Scholar, and other relevant databases. Keywords and MeSH terms such as early detection, prostate cancer, screening, overdiagnosis, and overtreatment were employed to identify relevant articles. The search was not restricted by publication date, but was limited to articles published in English. Inclusion criteria comprised studies that directly addressed the topic. Exclusion criteria were applied to filter out articles that did not align with the focus of this review. Initial screening involved evaluating titles and abstracts for relevance. Subsequently, the full text of selected articles was thoroughly reviewed to determine eligibility for inclusion in the narrative review. Data from the included studies were extracted and organized. Findings from the included studies were synthesized to provide a comprehensive overview of the current knowledge. Common themes, trends, and gaps in the literature were identified and discussed.

## 3. Background

### 3.1. Insights into Prostate Cancer Mortality before the Screening Era

PCa in the absence of routine screening often remained asymptomatic until it reached an advanced stage. Consequently, tumors tended to be larger, more aggressive, and had often metastasized by the time of diagnosis. This aggressive nature significantly reduced the effectiveness of available treatments, leading to higher mortality rates, reaching up to one in three diagnosed cases [6,7].

In addition to the issue of late diagnosis, only open radical prostatectomy, radiation and hormonal therapies were among the few available treatments available. Radical prostatectomy often led to substantial side effects, including urinary incontinence and sexual dysfunction. Hormonal therapies, while sometimes effective initially, impacted patients’ quality of life and often resulted in the development of castration-resistant cancer over time. These limitations in available treatments meant that the disease’s progression could not be adequately halted, contributing to higher mortality rates and having a severe impact on the quality of life of patients. Late-stage diagnoses meant patients often faced debilitating symptoms, including bone pain, urinary retention or acute renal failure. Psychological distress, both for patients and their families, was common. Palliative care became a crucial component, but could not entirely alleviate the suffering associated with the disease [8,9,10].

Public awareness campaigns about PCa symptoms, risk factors, and the importance of regular check-ups were virtually non-existent. Men lacked the knowledge to recognize the early signs of PCa, and even if they did, the urgency of seeking medical attention was often underestimated [11]. Table 1 summarizes the most important factors related to PCa mortality during the pre-screening era.

Understanding these multifaceted challenges in the pre-screening era underscores the pivotal role of contemporary screening methods. Early detection through PSA tests and advancements in treatment modalities have significantly transformed the landscape of PCa management.

### 3.2. Discovery of PSA and Its Revolutionizing Impact on Prostate Cancer Screening

PSA stands as a transformative discovery in PCa diagnosis and treatment. Discovered in the 1970s, PSA is an enzyme produced by the prostate gland, aiding in the liquefaction of semen. However, its significance in cancer detection became apparent in the following decades, leading to a revolutionary shift in how prostate cancer is diagnosed and monitored. Dr. T. Ming Chu and his team isolated PSA from prostate tissue. Initially regarded as merely a biomarker for prostatic diseases, its potential in cancer diagnostics was not immediately recognized. It was Dr. Richard Ablin who discovered PSA’s presence in both normal and cancerous prostate tissues, laying the foundation for further research. In the 1980s, with technological advancements, scientists developed the PSA blood test. This test measures the levels of PSA in the bloodstream, and elevated PSA levels can indicate various prostate conditions such as cancer, but also benign prostatic hyperplasia, urinary tract infection or prostatitis, etc. The PSA test became widely adopted for prostate cancer screening, offering a minimally invasive method to detect potential malignancies. The introduction of PSA screening led to a significant increase in the early detection of prostate cancer. Unlike many other cancers, PCa, especially in its early stages, often produces no symptoms. PSA testing identified early disease, enabling timely interventions and potentially life-saving treatments [12,13].

### 3.3. Previous Trials and Their Outcomes: Navigating Complex Results

The Prostate, Lung, Colorectal, and Ovarian (PLCO) Cancer Screening Trial [14], the European Randomized Study of Screening for Prostate Cancer (ERSPC) [15] and the Göteborg trial [16,17,18] are pivotal milestones in the landscape of PCa screening research. A critical analysis of these trials provides essential insights into the complexities surrounding screening efficacy, mortality reduction, and the ethical challenges of overdiagnosis.

#### 3.3.1. The Prostate, Lung, Colorectal, and Ovarian (PLCO) Cancer Screening Trial: Insights and Implications

In 1993, a landmark study initiated in the United States was designed to evaluate the effectiveness of various cancer screening methods, including PCa. The trial’s comprehensive approach provided valuable insights into the complexities of cancer screening, with a specific focus on PCa. The PLCO trial, conducted over a span of nearly two decades, aimed to assess the impact of regular screening tests, including the PSA test and DRE, on reducing cancer mortality. The trial involved a large cohort of participants, making it one of the most extensive studies in cancer screening history. The trial enrolled 76,693 men aged 55–74 years between 1993 and 2001. Participants in the screening group received annual PSA testing for 6 years and annual DRE for 4 years, being referred to the urologist in case of high risk. After a 13-year follow-up, the relative PCa incidence was 1.12 (95% CI 1.07 to 1.17), and the relative risk of PCa death was 1.09 (95% CI 0.87 to 1.36) in the screening group compared to the control group. However, these results cannot be used to evaluate the effect of screening versus no screening due to several factors: nearly half of the enrolled men had undergone PSA testing before the study, eventually 90% of the control group participants were PSA-tested, and less than half of the men with elevated PSA levels underwent a prostate biopsy [14].

The outcomes related to PCa screening in the PLCO trial revealed a nuanced picture. The trial indicated that routine PSA screening, as conducted in the study, did not significantly reduce mortality due to PCa compared to the control group. These findings were pivotal and led to substantial debates within the medical community regarding the overall efficacy of PSA-based screening programs. The results of the PLCO trial had profound implications for PCa screening guidelines and public health policies. The findings highlighted the challenges of overdiagnosis, where PSA screening led to the detection of indolent, slow-growing tumors that might not have posed a significant threat during the patient’s lifetime. Consequently, many men were over-treated, often resulting in adverse effects such as impotence and incontinence. Additionally, the PLCO trial underscored the importance of informed decision-making. It became clear that the benefits of routine PSA screening need to be carefully weighed against the potential harms, emphasizing the need for shared decision-making processes between healthcare providers and patients. The PLCO trial findings significantly influenced the guidelines provided by various medical organizations [19,20,21].

#### 3.3.2. The European Randomized Study of Screening for Prostate Cancer (ERSPC): Balancing Optimism and Challenges

This study is a significant multi-country initiative that sheds light on the potential benefits and complexities of PCa screening. Spanning across multiple European nations, this study provided a more optimistic view of screening’s impact on reducing PCa mortality, yet it illuminated the ethical and practical dilemmas inherent in early detection efforts. The ERSPC demonstrated a relative reduction in PCa mortality among men invited to screening. These promising results suggested that regular screening could save lives by detecting and treating PCa at an earlier, more treatable stage. This positive outcome sparked hope within the medical community, reinforcing that widespread screening programs could substantially decrease PCa related deaths [15].

This study, initiated in 1993, varied in recruitment and randomization procedures across countries. It employed serum PSA as the primary screening test, with a screening interval of 4 years for most centers. After a median follow-up of 9 years involving 162,242 men, the screening group showed a 20% reduction in prostate cancer mortality (rate ratio: 0.80, 95% CI 0.65 to 0.98). This equated to 0.71 fewer PCa deaths per 1000 men, but with an excess incidence of 34 PCa cases per 1000 men. The numbers of invitations and diagnoses needed to prevent one PCa death were 1410 and 48, respectively. Longer follow-ups revealed an increased reduction in mortality and decreased numbers of invitation and diagnoses required. A 12-year follow-up indicated a 50% reduction in metastatic disease at diagnosis and a 30% reduction overall. An analysis accounting for non-compliance and PSA testing in the control group demonstrated a net mortality reduction of 51% among screening participants (intention-to-screen analysis: 32%) [15,22,23].

However, beneath this optimism lay significant challenges. The ERSPC findings revealed the complexities associated with overdiagnosis and overtreatment. The ERSPC results brought forth ethical dilemmas that healthcare providers, researchers, and patients grappled with. Detecting tumors that might not pose an immediate threat raised the importance of new treatment options such as active surveillance. Informed decision-making became paramount, emphasizing the need for patients to fully understand the implications of screening, including the risks of overdiagnosis and the potential consequences of unnecessary treatments. Striking a balance between optimism for early detection and the ethical considerations of avoiding overdiagnosis and overtreatment became a focal point. The ERSPC findings underscored the importance of developing clear guidelines and communication strategies. Healthcare providers need to engage patients in open, honest discussions about the benefits and risks of screening and potential treatment options, ensuring that individuals could make decisions aligned with their values and preferences [3].

#### 3.3.3. The Göteborg Randomized Population-based Prostate Cancer Screening Trial

Initiated in Sweden in 1995, this trial involved a large cohort of men residing in Göteborg. The trial included 20,000 men aged 50–64 years, randomly assigned to biennial PSA screening (threshold 3 ng/mL for systematic biopsy) or a control group. Despite the fact that more than half of the control group had undergone PSA testing (i.e., 72% had at least 1 test), this trial exhibited the most significant reduction in PCa mortality among screening studies. After 14 years, the trial showed a 44% relative reduction in mortality (95% CI 28% to 64%), lowering absolute mortality from 0.9% to 0.5% (difference 0.4%, 95% CI 0.17% to 0.64%). After 22 years, the relative reduction was 29% (95% CI 9.0% to 0.45%), with an absolute reduction of 0.6% (95% CI 0.15% to 1.0%). Screening initiation at ages 50–55 and lower education level correlated with higher mortality reduction. The number of screenings needed to diagnose and prevent one PCa death was 12 at 14 years and 9 at 22 years. Only 0.6% of men with moderately elevated PSA (3–9.9 ng/mL) and negative first biopsy died from PCa within 20 years. Most PCa deaths in the screening group occurred in men who began screening after 60, did not attend regularly, or were diagnosed post-screening cessation. The protective effect of screening diminished 10–12 years after cessation. Even after 24 years, the control group’s PCa incidence did not reach that of the screening group, indicating that many cancers detected through screening might never have been clinically diagnosed [16,17,18].

Table 2 shows the most important differences between the three studies, highlighting the degree of contamination and the extra-diagnosis rate.

#### 3.3.4. New Technologies, Improved Strategies and New Risk-Stratified Screening Algorithms

Despite the diversity of the tools at our disposal, there will always be debate in terms of cancer detection rates and test availability, capacity, and costs, since we do not have the perfect risk stratification widget. Based on the existing literature and expert consensus opinion, a risk-stratified strategy was submitted for an early detection program for PCa that adequately assesses the trade-offs between overdiagnosis and underdetection [24]. Many approaches have been considered to enhance PCa screening, such as multivariable risk prediction models that take into account multiple variables, such as age, family history, race, PSA levels, and other biomarkers; these models aim to provide a more personalized risk assessment to guide decision making for both patients and clinicians.

“PSA-only”-based screening is considered an outdated strategy, due to the emergence of new diagnostic techniques. The advent of magnetic resonance imaging (MRI), risk calculators (RCs), novel blood-based and urine-based biomarkers and increased knowledge on the natural course of different risk groups improved the individual balance between the harms and benefits of early detection. Prostate MRI has gained attention as a tool to improve the accuracy of PCa detection. Multiparametric MRI combines different imaging techniques, offering detailed images of the prostate, potentially helping to identify clinically significant cancers and avoid unnecessary biopsies. Performing multiparametric MRI before biopsy is strongly recommended by the EAU guidelines. It not only has preferable detection rates, but could also reduce the number of biopsy procedures when MRI-negative men are excluded from prostate biopsy. In this way, some studies have found that the exclusion of men with PI-RADS 3 lesions and a PSA density of <0.13–0.15 ng/mL/cc is a safe strategy [24].

New blood-based biomarkers that showed good discriminative ability concerning the detection of csPCa are the Prostate Health Index (PHI) and the Four-Kallikrein Panel (4 K score). They contain basic and new PSA parameters (e.g., free PSA, total PSA, benign PSA, proPSA, and intact PSA). The urine-based biomarkers that have been developed are Prostate Cancer Antigen 3 (PCA3), TMPRSS2-ERG, and urinary three-gene pane (HOXC6, TDRD, and DLX) [25]. Unfortunately, head-to-head comparisons and validation studies are needed to determine which biomarkers are preferred. Genetic and molecular markers are being investigated to identify individuals at higher risk of developing aggressive forms of prostate cancer. Genetic testing may help in risk stratification and guide decisions regarding the intensity of screening and surveillance.

Regarding risk calculators, the variables commonly incorporated are PSA, digital rectal examination (DRE), age, %free PSA, transrectal ultrasound (TRUS), previous biopsy status, MRI result and PSA density, the latter being one of the strongest predictors among the RCs [26].

In 2021, Balndala-Jacques A. et al. [27] published a systematic review of prostate risk calculators at that moment, with the most commonly analyzed being the Prostate Cancer Prevention Trial (PCPT) and the European Randomized Study on Prostate Cancer (ERSPC) risk calculators. They concluded that both the PCPR and ERSPC risk calculators have been successfully adapted for cohorts other than the ones they were originally created for with no loss of diagnostic ability, which determines their ease of application and reproducibility.

Artificial intelligence (AI) applications tools, including machine learning algorithms, are being explored to analyze complex datasets and improve risk prediction in PCa. These technologies may assist in interpreting imaging results, genetic data, and other relevant information.

The implementation of any new screening algorithms requires thorough validation through clinical trials and ongoing research. Additionally, the balance between early detection and avoiding overdiagnosis/overtreatment remains a challenge in PCa screening.

### 3.4. The European Union’s Recommendation on PCa Early Detection

In March 2022, this evidence base led the Science Advice for Policy by European Academies (SAPEA) to publish an updated evidence review report [28] informing the European Commission (EC) Group of Chief Scientific Advisors on the latest available scientific developments in the landscape of PCa early detection. Based on this report, in September 2022, the EC submitted to the Council of the European Union (EU) a proposal, i.e., a new Council Recommendation for a new EU approach to cancer screening [29] (‘Council Recommendation on strengthening prevention through early detection: A new EU approach on cancer screening replacing Council Recommendation 2003/878/EC’). Considering the new evidence and latest technological innovations, the new approach calls for the extension of screening programs to other cancer sites including the prostate in a stepwise manner. On 9 December 2022, the Ministers of Health from all 27 EU member states came to an agreement, after which the Council of the EU adopted the new approach on cancer screening proposed by the EC. The recommendation invites member states to evaluate the feasibility and effectiveness of organized prostate cancer screening for men, based on prostate-specific antigen (PSA) testing combined with magnetic resonance imaging (MRI) scanning as a follow-up.

### 3.5. PRAISE-U Project

In line with this recommendation, the ‘PRostate cancer Awareness and Initiative in the EU’ (PRAISE-U) consortium was formed [30,31]. The PRAISE-U Project, which is led by the European Association of Urology (EAU), is dedicated to reducing morbidity and mortality caused by prostate cancer in the EU. This project aims to provide concrete evidence on a risk-stratified approach to the early detection of prostate cancer, also referred to as ‘smart early detection’.

#### 3.5.1. Aims and Methods

PRAISE-U’s overall goal is to align smart early detection protocols and guidelines across EU member states, while facilitating the collection and distribution of relevant data. This enables faster knowledge transfer and intends to fill knowledge gaps. The EAU and its partners have been politically active in advocating for the early detection and diagnosis of PCa through customized and risk-based population-based screening programs.

The EU has previously provided advice on screening for breast, cervical and colorectal cancer, as shown in projects such as CanScreen 5 and EU-TOPIA. The PRAISE-U consortium aims to build on the knowledge gained from these other cancer indications, including implementing tools such as the systematic methodology of quality assurance composed by the European Commission’s Joint Research Centre (JRC), and applying this to PCa.

#### 3.5.2. Work Packages and Consortium

**Figure 1:**
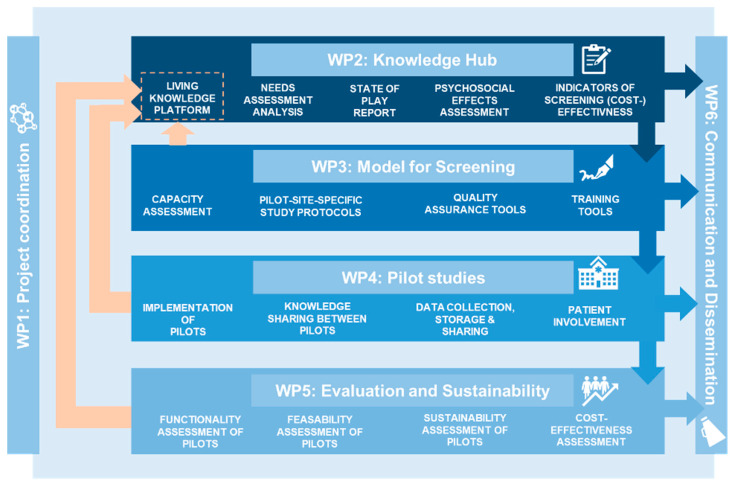
Work packages. PRAISE-U project.

**Figure 2:**
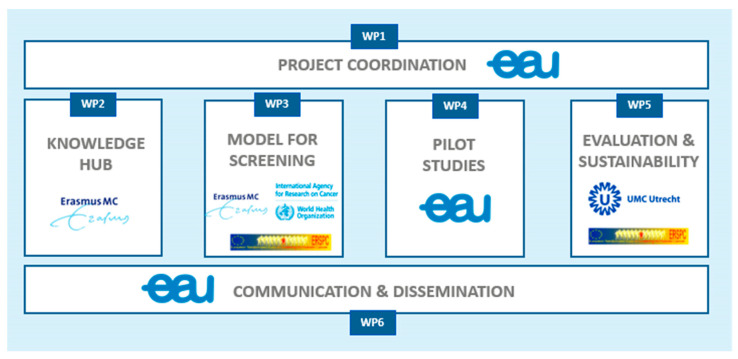
Work package leads. PRAISE-U project.

The project comprises six work packages (WPs), with each WP building on the work of the previous one. Four core WPs (WP2-5) focus on knowledge gathering (needs assessment and state of play report), the development of a site-specific implementation protocol based on health system capacity, pilot testing of these protocols and the evaluation of the results (Figure 1). Two overarching WPs (WP1,6) oversee project coordination and result dissemination. The project’s multidisciplinary consortium comprises 25 institutions from twelve member states, including leading clinicians and researchers, various field experts, a network of hospitals, medical societies, patient advocates and national authorities. Their responsibilities lie within the coordination of the joint action, screening efforts, and contribution of data from the pilot sites (Figure 2).

#### 3.5.3. Pilot Sites

The developed screening models will be pilot tested in five different pilot sites in Poland, Ireland, Spain (Galicia region), Spain (Manresa) and Lithuania. Each site is carefully chosen to represent different member state characteristics, each with a corresponding primary focus.

There are differences in terms of the target in each of the pilot sites.

- The Polish pilot explores the establishment of a screening program when infrastructure is limited. It promotes a hospital-based approach.
- The Irish pilot focuses on the streamlining of opportunistic testing within suggested screening intervals. It promotes home-based PSA testing.
- The Galician pilot assesses the feasibility of a risk-based approach, including psychosocial effects.
- The Manresa pilot focuses on compliance, particularly when primary healthcare providers are involved in the invitation process.
- In Lithuania, a PSA-based population screening trial with certain risk stratification is already in place. Thus, this pilot focuses on the alignment of the algorithm proposed by the PRAISE-U project and the formalization of its invitation system.

In Galicia, as well as in Manresa and Lithuania, global population screening is promoted.

The socioeconomic differences of the selected countries make the learning process even greater, considering that these are countries with different health systems, which will help us to implement these recommendations on a larger scale.

### 3.6. Implications for the Future PCa Screening Landscape

The landscape of PCa screening has undergone transformative shifts, particularly with the advent of screening initiatives endorsed by the EU. Screening is a powerful tool that can save lives and reduce the cancer burden. However, any population-wide cancer screening program—whether current or future—must be effective, equitable and cost-effective to maintain an optimal balance of benefits and harms [24]. Despite the evidence of the benefits of PSA-based screening trials being convincing, clinical trials cannot tell us exactly what will happen when a screening program is implemented in the real world. Local—whether national or regional—factors such as demographics and health service capacity should be considered.

In addition, although the proposed new risk-based screening strategy is expected to optimize the benefits of population-based screening while reducing the risk of unnecessary biopsies, overdiagnosis and consequent overtreatment [5], it has not yet been analyzed prospectively. The PRAISE-U project aims to provide concrete evidence on this by piloting this risk-stratified screening approach across five member states.

Along with the project’s efforts, further research and continuous monitoring of ongoing research is needed to assess the possibilities of emerging technologies such as radiomics and artificial intelligence, as well as to identify the groups that will most benefit from screening to ensure that an appropriate balance of benefits and harms is maintained. Further research is needed to determine the optimal age for screening initiation and discontinuation and the optimal number of screening rounds and intervals. In the meantime, member states need to be aware of the fact that offering ad hoc PSA testing for men without symptoms leads to inequity and should be discouraged in order to reduce the risk of overdiagnosis and overtreatment, especially in older men. Therefore, moving towards the improved early detection of PCa in the EU, the continuous evaluation of benefits, harms and cost-effectiveness, and ongoing program optimization is essential [24].

## 4. Conclusions

The landscape of PCa screening has undergone transformative shifts, particularly with the advent of screening initiatives endorsed by the EU, creating a new strategy which optimizes the early detection of clinically significant cancers as well as mitigating the risk of overdiagnosis and overtreatment. PRAISE-U aims to encourage the early detection and diagnosis of PCa through customized and risk-based screening programs, facilitating the collection and distribution of relevant data while incorporating the learnings of the last three decades. Further studies will be needed to identify those patients who benefit most from screening and at what age.

## Figures and Tables

**Table 1:** Key factors for high prostate cancer mortality before the screening era.

Key Factors Associated with High Prostate Cancer Mortality before the Screening Era
Late-Stage Diagnosis and Aggressive Disease Limited Awareness and Education Absence of Systematic Screening: Limited Treatment Options and Their Impact Undiagnosed Cases and Missed Opportunities Socioeconomic Disparities Variability in Survival Rates Impact on Quality of Life

**Table 2:** Summary of the 3 largest pilot studies in prostate cancer screening.

PLCO	Göteborg	ERSPC
76,693 men	20,000 men	182,160 men
Age 55–74	Age 50–64	Age 55–70
No difference in PCa mortality	29% PCa mortality reduction	20% PCa mortality reduction
Upfront: 34% contamination During trial: 52% contamination Underpowered trial	Low contamination To avoid one man dying and suffering from Prostate cancer Screen: 221 Extra diagnoses: 9	Low contamination To avoid one man dying and suffering from Prostate cancer Screen: 570 Extra diagnoses: 18

## Data Availability

Data are contained within the article.

## Appendix A. The PRAISE-U Consortium

Hendrik Van Poppel (EAU), Sarah Collen (EAU), James N’Dow (EAU), Phillip Cornford (EAU), Juan Gómez Rivas (EAU), Monique Roobol-Bouts (EMC, ERSPCF), Katharina Beyer (EMC), Lionne Venderbos (EMC, ERSPCF), Jozien Helleman (EMC), Renée Leenen (EMC), Daan Nieboer (EMC), Pia Kirkegaard (CDR), Berit Andersen (CDR), Louise Dybdahl Pedersen (CDR), Mette Bach Larsen (CDR), Sofie Meyer Andersen (CDR), Grace McKinney (CDR), Karel Hejduk (UZIS), Ondřej Májek (UZIS), Ondřej Ngo (UZIS), Tomáš Vyskot (UZIS), Marcela Koudelková (UZIS), Roman Zachoval (UZIS, CUS), Renata Chloupkova (UZIS), Katerina Hejcmanova (UZIS), Roderick van den Bergh (UMCU), Peter-Paul Willemse (UMCU), Norbert Couespel (ECO), Riccardo Moschetti (ECO), Nora Lorenzo (ECO), Mike Morrissey (ECO), Richard Price (ECO), Enea Venegoni (ECO), Agnese Konusevska (ECO), Ana Mula (ECO), Dorota Dudek-Godeau (DCOPiH, NIZP), Malgorzata Krynicka (DCOPiH), Krzysztof Tupikowski (DCOPiH), Kataryzyna Hodyra-Stefaniak (DCOPiH), Monika Pajewska (NIZP), Aleksandra Czerw (NIZP), Andrzej Deptała (NIZP), Ángel Gómez Amorín (CSG), Silvia Suárez Luque (CSG), Carmen Durán Parrondo (CSG), Ana Marina Tarrazo Antelo (CSG), Josep Vilaseca (ALT, WONCA), Gemma Cuberas Borrós (ALT), Anna Arnau Bartés (ALT), Juan Pablo Salazar (ALT), Hector López Llauradó (ALT), Ola Bratt (VGR), Rebecka Godtman (VGR), Emil Järbur (VGR), Thomas Jiborn (SKA), Anders Bjartell (SKA), Anna Holst (SKA), Max Alterbeck (SKA), Aušvydas Patašius (NCI), Gintare Miksiene (NCI), Adomas Ladukas (NCI), Giedrė Smailytė (NCI), Ernestas Janulionis (NCI), Zygimantas Kardelis (NCI), Marius Kincius (NCI), Mingaile Drevinskaite (NCI), Auguste Kaceniene (NCI), Lieven Annemans (UG), Pieter-Jan Hutsebaut (UG), Pieter Vynckier (UG), Robert Kidd (HSE), Michael O Brien (HSE), Paula Keon (HSE), Carolyne Lynch (HSE), Michael Rooney (HSE), Martin Kivi (EUS), David Galvin (UCD), Eamonn Rogers (UCD), Eileen Nolan (UCD), Paul Seeney (UCD), Alexander Bauer (WONCA), Thomas Frese (WONCA), Christine Bruetting (WONCA), Cate Bennett (MOV), Amy O’Connor (MOV), Sarah Coghlan (MOV), Ricky Le Roux (MOV), Karen Robb (MOV), Partha Basu (IARC), Arunah Chandran (IARC), Andre Carvalho (IARC), Deependra Singh (IARC), Lobna Boulegroun (IARC), Milagros Otero-García (ESUR), Erik Briers (Europa UOMO), Anna Lantz (RS), Lisa Jelf Eneqvist (RS)

STICHTING EUROPEAN UROLOGICAL FOUNDATION (EAU), established in Mr. E.N. van Kleffensstraat 5, 6842 CV Arnhem, Netherlands;

ERASMUS UNIVERSITAIR MEDISCH CENTRUM ROTTERDAM (EMC), established in Dr Molewaterplein 40, Rotterdam 3015 GD, Netherlands;

STICHTING EUROPESE STUDIE PROSTAATKANKER SCREENING (ERSPCF), established in Schaarweide 5, Reeuwijk 2811 JM, Netherlands;

REGION MIDTJYLLAND (CDR), established in Skottenborg 26, Viborg 8800, Denmark;

USTAV ZDRAVOTNICKYCH INFORMACI A STATISTIKY CESKE REPUBLIKY (UZIS), established in Palackeho Namesti 4, Praha 12801, Czechia;

UNIVERSITAIR MEDISCH CENTRUM UTRECHT (UMCU), established in Heidelberglaan 100, Utrecht 3584 CX, Netherlands;

EUROPEAN CANCER ORGANISATION (ECO), established in Rue De La Science 41, Bruxelles 1040, Belgium;

DOLNOSLASKIE CENTRUM ONKOLOGII, PULMONOLOGII I HEMATOLOGII (DCOPiH), established in Ul. Pl. Ludwika Hirszfelda, 12 000, 53-413, Wroclaw, Poland;

NARODOWY INSTYTUT ZDROWIA PUBLICZNEGO PZH–PANSTWOWY INSTYTUT BADAWCZY (NIZP), established in Chocimska 24, Warszawa 00791, Poland;

CONSELLERIA DE SANIDADE DE GALICIA (CSG), established in Edificio Administravito San Lazaro sn, Santiago de Compostela 15781, Spain;

ALTHAIA XARXA ASSISTENCIAL UNIVERSITARIA DE MANRESA, FUNDACIO PRIVADA (ALT), established in Calle Doctor Joan Soler 1–3, Manresa, Barcelona 08243, Spain;

VASTRA GOTALANDSREGIONEN (VGR), established in Regionens Hus, Vanersborg 462 80, Sweden;

REGION SKANE (SKA), established in Region Skane, Kristianstad 291 89, Sweden;

NACIONALINIS VEZIO INSTITUTAS (NCI), established in Santariskiu Str, 1, Vilnius 08660, Lithuania;

GHENT UNIVERSITY (UG), public institution with legal personality, having its administrative offices at Sint-Pietersnieuwstraat 25, B-9000 Gent, Belgium;

HEALTH SERVICE EXECUTIVE (HSE), established in Limetree Avenue, Millenium Park, NAAS Nass, County Kildare, Ireland;

EESTI UROLOOGIDE SELTS (EUS), established in L Puusepa TN 8, Tartu 51014, Estonia;

UNIVERSITY COLLEGE DUBLIN, NATIONAL UNIVERSITY OF IRELAND, DUBLIN (UCD), established in Belfield, Dublin 4, Ireland;

STICHTING WONCA EUROPE (WONCA), established in Poljanski Nasip 58, Ljubljana 1000, Slovenia;

MOVEMBER FOUNDATION EV (MOV), established in Leopoldstr 11 A, Munchen 80802, Germany;

INTERNATIONAL AGENCY FOR RESEARCH ON CANCER (IARC), the cancer research agency of the World Health Organization (WHO), whose offices are at 25 avenue Tony Garnier, CS 90627, 69366 LYON CEDEX 07, France

EUROPEAN SOCIETY OF UROGENITAL RADIOLOGY (ESUR), whose administrative offices are at Landstrasser Hauptstrasse 27, Eingang Weyrgasse 9 / Tür 15, 1030, Vienna, Austria;

EUROPA UOMA (Europa UOMO), whose administrative offices are atLeopoldstraat 34 000, 2000, Antwerpen, Belgium;

THE CZECH UROLOGICAL SOCIETY (EUS), whose administrative offices are at Sokolská 490/31, 120 00, Praha 2, Czechia;

REGION STOCKHOLM (RS), whose administrative offices are at Hantverkargatan 45 22550, 104 22, Stockholm, Sweden;
